# Three-nucleotide periodicity of nucleotide diversity in a population enables the identification of open reading frames

**DOI:** 10.1093/bib/bbac210

**Published:** 2022-06-13

**Authors:** Mengyun Jiang, Weidong Ning, Shishi Wu, Xingwei Wang, Kun Zhu, Aomei Li, Yongyao Li, Shifeng Cheng, Bo Song

**Affiliations:** Chinese Academy of Agricultural Sciences and Henan University, China; Chinese Academy of Agricultural Sciences and Huazhong Agricultural University, China; Chinese Academy of Agricultural Sciences and Henan University, China; Chinese Academy of Agricultural Sciences and Henan University, China; Chinese Academy of Agricultural Sciences and Henan University, China; Chinese Academy of Agricultural Sciences, China; Chinese Academy of Agricultural Sciences, China; Chinese Academy of Agricultural Sciences, China; Chinese Academy of Agricultural Sciences, China

**Keywords:** open reading frame, sORF, SNPs, population, polyploidy genome

## Abstract

Accurate prediction of open reading frames (ORFs) is important for studying and using genome sequences. Ribosomes move along mRNA strands with a step of three nucleotides and datasets carrying this information can be used to predict ORFs. The ribosome-protected footprints (RPFs) feature a significant 3-nt periodicity on mRNAs and are powerful in predicting translating ORFs, including small ORFs (sORFs), but the application of RPFs is limited because they are too short to be accurately mapped in complex genomes. In this study, we found a significant 3-nt periodicity in the datasets of populational genomic variants in coding sequences, in which the nucleotide diversity increases every three nucleotides. We suggest that this feature can be used to predict ORFs and develop the Python package ‘OrfPP’, which recovers ~83% of the annotated ORFs in the tested genomes on average, independent of the population sizes and the complexity of the genomes. The novel ORFs, including sORFs, identified from single-nucleotide polymorphisms are supported by protein mass spectrometry evidence comparable to that of the annotated ORFs. The application of OrfPP to tetraploid cotton and hexaploid wheat genomes successfully identified 76.17% and 87.43% of the annotated ORFs in the genomes, respectively, as well as 4704 sORFs, including 1182 upstream and 2110 downstream ORFs in cotton and 5025 sORFs, including 232 upstream and 234 downstream ORFs in wheat. Overall, we propose an alternative and supplementary approach for ORF prediction that can extend the studies of sORFs to more complex genomes.

## Introduction

Annotation of open reading frames (ORFs) in genomes is one of the most important processes required for downstream analyses and the use of reference genomes. Various algorithms have been developed to predict ORFs [1–5] in genomes, but these sequence-based methods are powerless in predicting small/short ORFs (sORFs) because many nonsense sORFs can arise by chance as random combinations of nucleotides. Recent studies have shown the crucial roles of sORFs, which encode peptides shorter than 100 amino acids, in various biological processes, including responses to abiotic and biotic stresses in plants [6] and oncogenesis in humans [7] and some of them are pertinent to cancer therapy [8]. The prediction of sORFs has long been problematic due to their short lengths and the use of alternative start codons, such as near-cognate codons (CUG, GUG, UUG) [4, 9]. Early attempts at sORF prediction were based on the sequence similarity across near or distant species, assuming that the functional sORFs would be conserved in sequence. For example, 26 conserved upstream ORFs (uORFs) were identified by comparing the full-length complementary DNA (cDNA) sequences between rice and *Arabidopsis* [10]. The recent application of the Ribo-Seq technique, which profiles ribosome-protected messenger RNA (mRNA) footprints (RPFs), has enabled accurate prediction of translated ORFs, including sORFs, in several genomes [4, 6, 8, 11–16]. Protected by a single ribosome sliding along the mRNA strand with a consistent step size of 3-nt, the RPFs should be uniform in size and show a 3-nt periodicity along the mRNAs if they are properly prepared [2], allowing the identification of the translating frame on mRNAs.

Although the Ribo-seq technique has been used in studies of many species, including yeast, humans, animals and plants [16], most of these species are model organisms with a simple genome. This is because a typical eukaryotic ribosome footprint is 28 nt in length [2, 17, 18], which is too short for accurate mapping in genome sequences, and this problem would be even worse in polyploid genomes. Due to multiple duplication events during evolutionary history, many plant genomes are polyploid and complex, featuring high repetitiveness and high heterozygosity [19, 20], limiting the application of Ribo-Seq in many plants, including several staple crops, such as bread wheat (*Triticum aestivum*) (6X) and cotton (*Gossypium hirsutum*) (4X). In addition, the challenges in preparing high-quality RPFs also limit the application of this technique to a broader range of non-model organisms. Incomplete digestion of unprotected mRNAs can reduce or eliminate the 3-nt periodicity of RPFs [21, 22], becoming useless for ORF identification.

Although the 3-nt periodicity shown by high-quality RPFs is powerful in ORF prediction, the application of RPFs for this purpose is hampered by the complexity of genomes, particularly in plant studies. We noticed that the populational diversity of nucleotides in coding sequences (CDSs) also shows a significant 3-nt periodicity, as observed for high-quality RPFs, and thus propose to use this periodicity to predict ORFs. In this study, to achieve this, we developed a Python package, ORF predictor using population genomic dataset (OrfPP), which identifies ORFs using the nucleotide diversities in populational datasets of single-nucleotide polymorphisms (SNPs). We tested OrfPP and recovered an average of 83.20% of the annotated ORFs with an average accuracy of 94.77%. The performance of OrfPP is robust even when a small subset of the SNPs is used. Finally, we applied OrfPP to two polyploidy genomes, cotton and wheat and identified 4704 and 5025 novel ORFs, respectively, with reliability comparable to the annotated ORFs in the genomes. Our study suggests that predicting ORFs from SNPs can be an approach supplementary to the existing methods and can be used to identify sORFs in complex genomes, for which the existing methods are not yet workable. We believe that this approach will play a greater role in future studies of animals and plants, given the rapidly growing number of SNP datasets for different species and the application of advanced techniques of DNA sequencing and SNP calling.

## Methods and datasets

### Comparison of ORFs predicted from RPFs and SNPs

To simplify the comparisons, the RPF-based ORF prediction in all the tested datasets was performed using RiboCode (v1.2.11) [23], ORFquant [24] and Ribotricer [25] with default parameters, and the ORFs predicted by the one performing the best (measured by *F*-score) were chosen to represent the ORFs predicted from RPFs. The predicted ORFs identical to the annotated ORFs in the reference genome were counted as true positives and the others were considered false positives. The accuracy, recall and F-score were calculated following the formula:

(1) \begin{equation*} \mathrm{Accuracy}=\frac{\mathrm{Number}\ \mathrm{of}\ \mathrm{true}\ \mathrm{positives}}{\mathrm{Total}\ \mathrm{number}\ \mathrm{of}\ \mathrm{predicted}\ \mathrm{ORFs}} \end{equation*}

(2) \begin{equation*} \mathrm{Recall}=\frac{\mathrm{Number}\ \mathrm{of}\ \mathrm{true}\ \mathrm{positives}}{\mathrm{Total}\ \mathrm{number}\ \mathrm{of}\ \mathrm{annotated}\ \mathrm{ORFs}} \end{equation*}

(3) \begin{equation*} \mathrm{F}-\mathrm{score}=\frac{2\times \mathrm{Recall}\times \mathrm{Accuracy}}{\mathrm{Recall}+\mathrm{Accuracy}}. \end{equation*}

F-scores were used to comprehensively assess the performance of OrfPP. It should be noted that although the unannotated ORFs were counted as false positives, many of them are *bona fide* ORFs, such as sORFs that were not included in the annotation of reference genomes.

### Metagene analysis and periodicity assessment

Metagene analyses have been used to illustrate the read distribution pattern of RPFs on mRNAs, which show a clear 3-nt periodicity in various organisms [9, 11–13, 26]. To perform metagene analysis of CDSs, the diversity for each nucleotide in the first 50 nucleotides was determined for each ORF and used to calculate the average nucleotide diversity at each position in the first 50 nucleotides for all the ORFs in the genome. Similarly, the diversity for each nucleotide in the 50 nucleotides upstream of start codons was selected for the analysis of the 5′ untranslated region (UTR), and those downstream of stop codons were selected for analysis of the 3′ UTR. To analyse intergenic regions, 10 000 50 bp windows were randomly selected and aligned to calculate the average diversity for the nucleotides at each position. The periodicity in the plot of metagene analyses was evaluated using an *F test* implemented in the ‘multitaper’ R package (version 1.0–14) [27], which detects the spectra and frequencies of the changes of nucleotide diversities within the window and calculates the *P-*values for all the frequencies. As a frequency of 0.33 Hz (1/3) indicates the periodic appearance/increase of the diversity of every three nucleotides, a significant *P-*value is expected for a pattern with 3-nt periodicity.

### Quantification of translation levels of ORFs

The RPFs used for ORF prediction in this study were also used to calculate the translation levels of the predicted ORFs. Briefly, the RPFs were mapped to the genomes using HISAT2 [28] with default parameters and the number of mapped RPFs in each ORF was counted and used to calculate translation levels.

### Validation of predicted ORFs using mass spectrometry datasets

To validate the ORFs predicted by OrfPP, we downloaded the protein mass spectrometry (MS) datasets of *Schizosaccharomyces pombe*, *Arabidopsis thaliana* and *Oryza sativa* from the PRIDE archive under the accessions of PXD015484 (*S. pombe*) [29], PXD009484 (*A. thaliana*) [30], PXD019885 (*O. sativa*) [31], PXD018692 (*G. hirsutum*) [32] and PXD021446 (*T. aestivum*) [33]. The MS raw data derived from the wild types of these species were downloaded and loaded into MaxQuant [34] with default parameters to search for the peptides encoded by the ORFs identified by OrfPP from SNPs. The percentage of ORFs supported by MS evidence was calculated for each class of ORFs, followed by normalization by their average expression levels (because ORFs with higher expression levels have a greater chance of being included in MS data) and log transformation. Therefore, the degree of MS support was calculated following the formula:

Degree of MS support = $\mathit{\log}10\Big(\frac{\mathrm{Number}\ \mathrm{of}\ \mathrm{ORFs}\ \mathrm{represented}\ \mathrm{in}\ \mathrm{MS}}{\mathrm{Total}\ \mathrm{number}\ \mathrm{of}\ \mathrm{ORFs}\times \mathrm{Expression}\ \mathrm{level}\ \mathrm{of}\ \mathrm{ORFs}}\Big).\quad $(4)

### Datasets

Several datasets from model organisms, including fission yeast, *Arabidopsis* and rice, were selected to test the performance of OrfPP. These organisms usually have high-quality reference genomes, SNP datasets [35–39] and high-quality RPFs [11, 26], allowing a comprehensive comparison between the ORFs predicted from RPFs and those predicted from SNPs by OrfPP. SNP datasets of cotton and wheat were also used to identify novel ORFs in the genome. The datasets used in this study are listed in detail in Table S1 (see Supplementary Data available online at http://bib.oxfordjournals.org/).

## Results

### 3-nt periodicity of nucleotide diversity in coding regions

**Figure 1:**
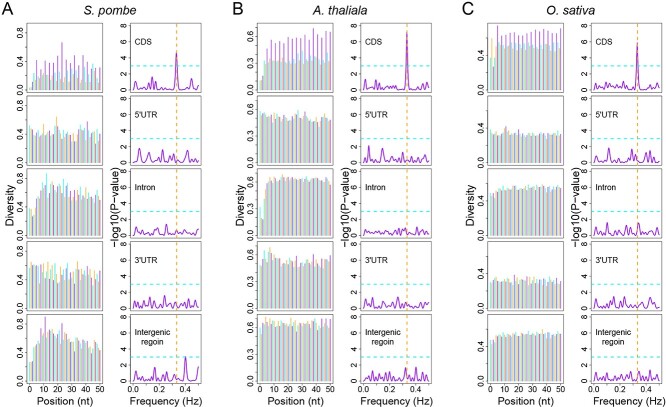
A 3-nt periodicity is shown by nucleotide diversity in coding sequences but not in the other regions in the genomes of (**A**) fission yeast, (**B**) *Arabidopsis* and (**C**) rice. The periodicity of the nucleotide diversities in each dataset was measured by a ‘multitaper’ test shown on the right, in which a peak at 0.33 (blue dashed lines) indicates a significant (*P* < 0.001, cyan dashed lines) periodicity of 3-nt. The values from the first, second and third positions in each triplet were colored in cyan, orange and purple, respectively.

**Figure 2:**
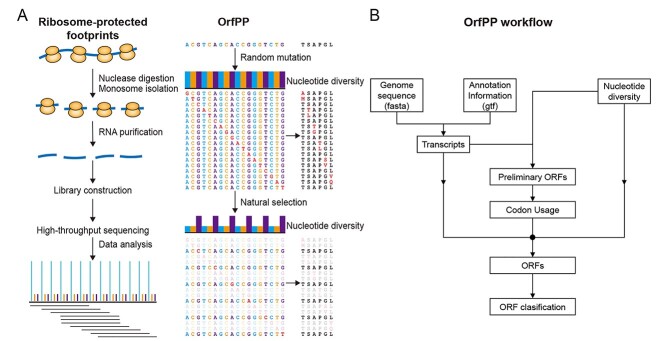
The workflow of OrfPP. (**A**) The 3-nt periodicity shown in the populational nucleotide diversity in CDSs is reminiscent of the periodicity shown in ribosome-protected footprints and (**B**) the workflow of OrfPP.

We performed metagene analyses of sequences from intergenic regions, 5′ and 3′ UTRs, and coding regions in different genomes ranging from yeast to higher plants (*Arabidopsis* and rice), the population size of which varied from 148 (yeast) to as large as ~3000 in rice. Our results show highly consistent patterns for all these tested populations; only the nucleotide diversities in CDSs showed a significant 3-nt periodicity, while the diversities in other regions appeared to vary randomly (Figure 1). The wobble nucleotides in codons are more tolerant to mutation and have experienced less purification selection during evolution. Indeed, higher diversities were observed for the third nucleotides in codons (Figure 1). In line with this observation, the nucleotide diversities in the non-coding regions were generally higher than those in CDSs, with those in intergenic regions being the highest, indicative of the smallest selective pressure on these regions. These results suggest that this 3-nt periodicity of nucleotide diversity in CDSs is common in natural populations. Such a periodic increase in nucleotide diversity in CDSs is reminiscent of the 3-nt periodicity of RPFs’ depth on mRNAs (Figure 2A); therefore, we propose that this periodicity of nucleotide diversity can also be used to predict ORFs.

### Design of OrfPP

To utilize the periodicity of nucleotide diversity to predict ORFs in genomes, we developed a pipeline named ‘OrfPP’, which is available as a Python package (https://pypi.org/project/OrfPP/1.0/). Given that the 3-nt periodicity of nucleotide diversity only appears in CDSs, as shown in Figure 1, it can distinguish CDSs from non-CDSs, determine the reading frame of ORFs and predict ORFs in the genome. Codon usage is also considered in OrfPP. The use of different codons varies considerably across different genomes, which are strongly correlated to the abundance of their corresponding tRNAs in the genome [40–42] and, therefore, is an intrinsic feature unique to each genome. It is reasonable to assume that all the ORFs in a genome use codons with the same preferences because they all share the same pool of tRNAs.

Briefly, OrfPP uses the 3-nt periodicity of nucleotide diversity in populational genomic datasets and codon usage preferences to predict ORFs in the genome. It takes three input files: the nucleotide diversity (derived from the SNP dataset), the reference genome sequence (in fasta format) and genome annotation (in gtf format) and includes three major steps, as shown in Figure 2B.

#### Codon usage training

To calculate genome-wide codon usages, OrfPP first predicts ORFs solely based on nucleotide diversity. The pipeline extracts the transcripts according to the genome annotation and allocates the values of nucleotide diversity onto each position on transcripts, from which candidate ORFs (start with ‘AUG’ and end with stop codons with a length multiple of three) are extracted for the prediction of ORFs. Two Student’s *t*-tests are performed to test whether the nucleotide diversities in frame 2 (the third nucleotides of codons) are greater than those in frame 0 and frame 1 in OrfPP, and are combined to report a final *P-*value for the identification of ORFs. To obtain ORFs with higher reliability for codon usage training, several stringent criteria are applied in this process of preliminary prediction. For example, at this stage, OrfPP only predicts ORFs starting with the canonical initiation codon ‘AUG’ from the candidates longer than 300 bp. The codon usages are then calculated from these predicted ORFs and used to represent the genome-wide usages.

#### ORF prediction

Transcript sequences are extracted according to the genome annotation information, and candidate ORFs were retained (Figure 2B) for further tests. In addition to the tests of nucleotide diversity between the frames (frame 0 versus frame 2, frame 1 versus frame 2), the pipeline also tests the codon usages between the frames for each candidate ORF by assigning to each nucleotide a value corresponding to the usage of the corresponding triplet as a codon in the genome. As for the diversity tests, OrfPP tests whether the values of codon usage at frame 0 are higher than those at frames 1 and 2 and combines the *P-*values from these four Student’s *t*-tests to give a final *P-*value for the prediction of ORFs. To also report sORFs, OrfPP reports ORFs shorter than 100 bp and the initiation codons can be customized.

#### ORF classification

The predicted ORFs are classified into 11 categories following previous definitions [1, 3, 5]. They are (i) annotated ORF, (ii) truncated ORF, (iii) extended ORF, (iv) uORF, (v) overlapped uORF, (vi) downstream ORF (dORF), (vii) overlapped dORF, (viii) ORFs located in non-coding RNAs, (ix) internal ORF, (x) ORFs located in transposable elements and (xi) ORFs in pseudogenes [3]. Although the ORFs are classified according to the previous annotation of ORFs in the reference genome, OrfPP can also be used for *de novo* prediction of ORFs when the annotation of ORFs is not yet known. In this situation, the predicted ORFs will all be annotated as ‘Novel ORFs’.

### Comparison of annotated ORFs recovered from RPFs and SNPs

Several species (Table S1, see Supplementary Data available online at http://bib.oxfordjournals.org/), including yeast, *Arabidopsis* and rice, all with high-quality reference genomes and available datasets of both RPFs and SNPs, were selected to assess the performance of OrfPP. The accuracy, recall and F-score of prediction were used to describe the performance of OrfPP in these genomes. We used the annotated ORFs in the reference genomes as a benchmark to measure the performance of OrfPP, with the predicted ORFs identical to the ORFs annotated in the reference genome considered true positives and false positives otherwise. A note of caution should be made that many unannotated ORFs may be *bona fide* ORFs, such as sORFs, not included in the annotation of reference genomes but considered false positives in this computation.

**Figure 3:**
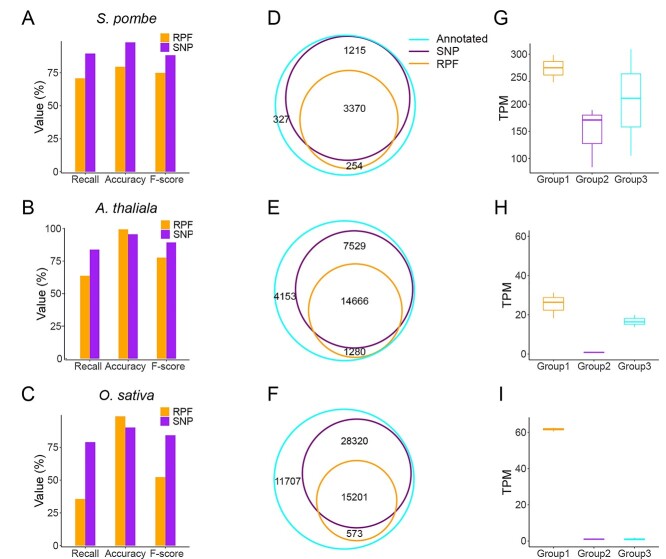
Recovery of annotated ORFs by OrfPP using SNPs datasets. Comparison between the ORFs predicted from SNPs and those from RPFs in the genomes of (**A**) fission yeast, (**B**) *Arabidopsis* and (**C**) rice. Overlaps between the ORFs predicted from SNPs (by OrfPP) and RPFs in (**D**) fission yeast, (**E**) *Arabidopsis* and (**F**) rice, according to which the annotated ORFs were categorized into three groups. The comparison of translation levels of genes between the three groups in (**G**) fission yeast, (**H**) *Arabidopsis* and (**I**) rice.

As RPFs were the most direct evidence recording the reading frame on the mRNAs, the ORFs predicted by OrfPP were also compared with those predicted from RPFs. RPF-based ORF prediction was performed using three different tools, RiboCode [5], ORFquant [24] and Ribotricer [25], the results of which varied substantially, with ORFquant performing the best in yeast datasets, while RiboCode was the best for *Arabidopsis* and rice (Table S2, see Supplementary Data available online at http://bib.oxfordjournals.org/; Figure S1, see Supplementary Data available online at http://bib.oxfordjournals.org/). We then used the yeast ORFs predicted by ORFquant and *Arabidopsis* and rice ORFs predicted by RiboCode to compare with those predicted from SNPs. Our analysis showed that 89.57, 83.82 and 79% of the known ORFs in the reference genome of fission yeast, *Arabidopsis* and rice were successfully recovered by OrfPP with accuracies of 98.03, 95.6 and 90.19%, respectively, from the SNP datasets. Many of the annotated ORFs in these genomes were also recovered from RPFs, but there were fewer than those found using SNPs by OrfPP (Figure 3A–C; Table S2, see Supplementary Data available online at http://bib.oxfordjournals.org/; Figure S1, see Supplementary Data available online at http://bib.oxfordjournals.org/), probably because the RPFs included only actively translating ORFs. RPFs from silenced ORFs under the tested conditions would have been absent in the RPF dataset. However, the accuracies of prediction are similar between these two tools for all the tested genomes, suggesting a comparable accuracy between the ORFs predicted from SNPs and those from RPFs. The comparison between the ORFs predicted from RPFs and those from SNPs indicates that most of the former were included by the latter (Figure 3D–F).

To further investigate the impacts of translation levels on the prediction of ORFs, we roughly categorized the known ORFs into three major groups: (i) commonly predicted from both RPFs and SNPs, (ii) predicted only from SNPs and (iii) not predicted, and calculated their translation levels using the corresponding RPFs used for ORF predictions in this study. Our data revealed the highest translation levels for the ORFs commonly predicted from both SNPs and RPFs (Figure 3G–I), suggesting that RPFs are efficient in predicting actively translated ORFs. However, the ORFs in the other two groups showed lower levels of translation (Figure 3G–I). These results suggest that in addition to the active ORFs that RPFs can capture, OrfPP can also predict inactive ORFs by utilizing the SNPs accumulated during the evolutionary history of the species.

### Comparison of sORFs identified from RPFs and SNPs

RPFs have been used to predict sORFs in many studies, but a shortcoming of this method has often been ignored. Due to the short size of RPFs, only a few of them can be uniquely mapped to genomes (Figure S2,see Supplementary Data available online at http://bib.oxfordjournals.org/). For example, for the RPFs tested in this study, the unique mapping rates of RPFs varied from 9 to 54%, while the unique mapping rates of whole-genome sequencing (WGS) reads can be as high as 92% (Figure S2, see Supplementary Data available online at http://bib.oxfordjournals.org/). The usual solutions to this multimapping problem are using only the uniquely mapped RPFs or randomly assigning the RPFs to one of the potential mapping sites [6, 11, 43, 44]. These solutions could potentially result in the misidentification of ORFs or missing some of the ORFs in the genome. Given the higher unique mapping rate of WGS reads, the sORFs predicted from SNPs are likely to be more complete than those from RPFs. Therefore, we also tested the performance of OrfPP in the identification of sORFs and compared those predicted from SNPs with those predicted from RPFs. In this comparison, to better illustrate the performance of OrfPP, we used the sORFs predicted from RPFs as a benchmark to assess the accuracy and recall rate of the predictions by OrfPP. The OrfPP-predicted sORFs identical to those predicted from RPFs were considered true positives and the others were tentatively considered false positives.

**Figure 4:**
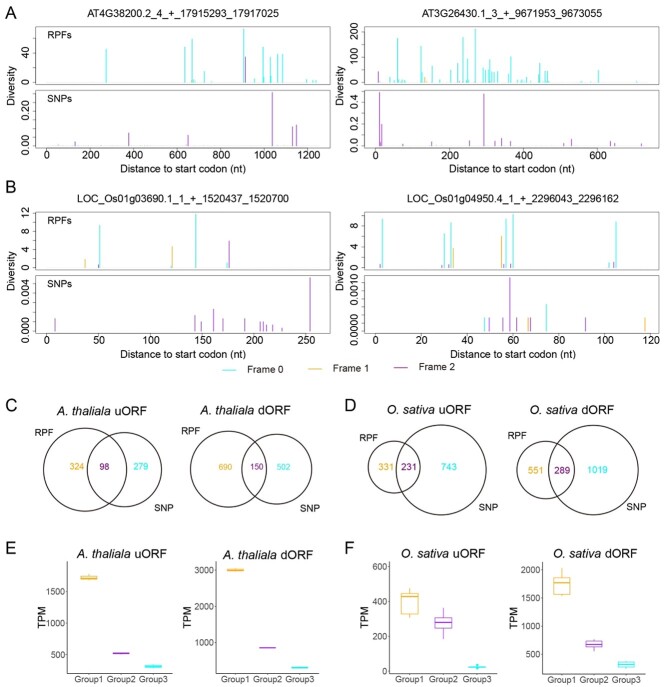
The prediction of sORFs in the genomes of *Arabidopsis* and rice. Examples of sORFs predicted from SNPs datasets of (**A**) *Arabidopsis* and (**B**) rice. The values from each triplet’s first, second and third positions were colored in cyan, orange and purple, respectively. Overlaps between the sORFs predicted from SNPs and RPFs in (**C**) *Arabidopsis* and (**D**) rice. Translation levels of the sORFs in different groups of (**E**) *Arabidopsis* and (**F**) rice.

In total, RPF-based tools predicted 72, 422 and 562 uORFs in the yeast, *Arabidopsis* and rice genomes, respectively (Figure 4A–D). However, only three uORFs were predicted by OrfPP from yeast SNPs, and none were identical to the uORFs predicted from RPFs. We found that the difference between these two prediction algorithms could be attributed to the difference in sORF definition in the tools. The yeast ORFs from RPFs were predicted by ORFquant, which reports sORFs as short as 9 bp, while OrfPP only reports ORFs longer than 60 bp. The other two RPF-based tools also report longer sORFs, but none of them predicted sORFs from yeast RPFs in this work. Therefore, the yeast sORFs predicted by ORFquant are not comparable to those predicted by OrfPP. For this reason, we compared only *Arabidopsis* and rice sORFs predicted from RPFs or SNPs. In total, 377 and 974 uORFs were predicted from *Arabidopsis* and rice SNPs and of these, 98 and 231 were identical to the uORFs predicted from RPFs, accounting for 23.22 and 41.10% of the total predictions from RPFs, respectively. Two ORFs commonly identified from RPFs and SNPs are shown for *Arabidopsis* (Figure 4A) and for rice (Figure 4B). These results suggest that more than a quarter of the uORFs predicted from RPFs can be recovered by OrfPP from SNPs. However, fewer dORFs were commonly identified from both SNPs and RPFs (Figure 4). Among the 840 dORFs identified using *Arabidopsis* RPFs, 150 (17.86%) were also identified from SNPs by OrfPP; 289 of the 840 (34.4%) dORFs identified from rice RPFs were recovered by OrfPP from SNPs (Figure 4C and D). As above, we categorized the sORFs into three groups: (i) ORFs identified from both SNPs and RPFs, (ii) ORFs identified from only RPFs and (iii) ORFs identified only from SNPs. Our data showed that the ORFs commonly identified from both the SNPs and RPFs were more actively translated. In contrast, the ORFs in the other two groups, particularly the ORFs in group (iii), identified from SNPs but not from RPFs, had lower translation levels (Figure 4E and F). The difference between these two predictions suggests a rare translation of dORFs but implies a potential to encode micropeptides in the 3′ UTR regions.

### ORF prediction from SNPs of polyploidy genomes

**Figure 5:**
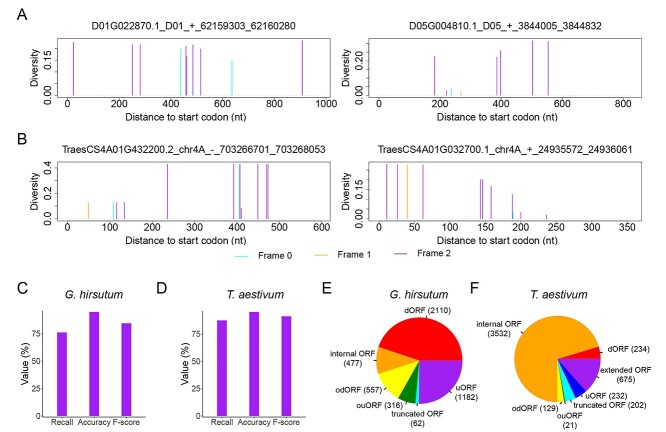
Application of OrfPP in complex genomes. Examples of identified ORFs from (**A**) cotton and (**B**) wheat. Performance of OrfPP in ORF identification from (**C**) cotton and (**D**) wheat SNPs. Novel ORFs identified from SNPs of (**E**) cotton and (**F**) wheat.

We further applied OrfPP to two polyploid genomes, tetraploid cotton (*G. hirsutum*) and hexaploid wheat (*T. aestivum*), to test its performance in complex genomes. SNP datasets derived from either 1913 cotton accessions [45] or 507 wheat accessions [46, 47] were used in this test. Both datasets showed a significant 3-nt periodicity in CDSs (Figure S3, see Supplementary Data available online at http://bib.oxfordjournals.org/), which allows the identification of ORFs. As a result, 91 594 ORFs were identified from cotton SNPs, among which 86 890 (94.86%) and 4704 (5.14%) were annotated and novel ORFs, respectively, and 114 929 ORFs, including 109 904 (95.63%) annotated and 5025 (4.37%) novel ORFs, were identified from wheat SNPs. The annotated ORFs recovered from SNPs accounted for 87.43 and 76.17% of all the annotated ORFs in the genome of cotton and wheat, respectively (Figure 5A–D), values comparable to the results using the model genomes (Figure 3). In addition to these annotated ORFs, several novel ORFs, including a variety of sORFs, were also identified from these datasets (Figure 5E and F), including 1182 and 2110 uORFs and dORFs, respectively, in the cotton genome and 232 and 234 uORFs and dORFs, respectively, in the wheat genome. These results suggest that ORF prediction from SNPs is also workable for complex genomes.

### Validation of novel ORFs identified from SNPs

**Figure 6:**
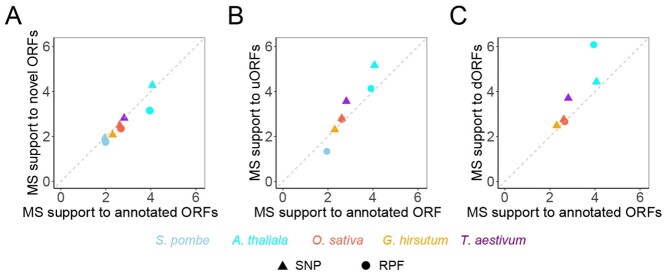
MS support to novel ORFs. Comparison of MS support between (**A**) all the novel ORFs, (**B**) uORFs and (**C**) dORFs and annotated ORFs identified from RPFs (circles) or SNPs (triangles) in different genomes.

Novel ORFs, including a variety of sORFs, were identified from both the RPF and SNP datasets in our tests. To verify the reliability of these predictions, we obtained protein MS data for each organism and searched for evidence supporting the existence of the peptides encoded by the novel ORFs. We computed the percentage of ORFs supported by MS data in each ORF class and, given the higher representation in MS datasets for ORFs with higher expression levels, measured the degree of MS support by normalizing this percentage to the average expression levels of ORFs in each class. Although more annotated ORFs were found in MS data compared to the novel ORFs, the degree of MS support for the annotated and novel ORFs predicted was generally comparable in our tests (Figure 6A). For the ORFs predicted from SNPs, the MS support for the novel ORFs is proportional to that of the annotated ORFs in the genome. Support values are near the diagonal of the plot (Figure 6A), suggesting equivalent reliability between these two categories of ORFs. Therefore, the novel ORFs predicted from SNPs could be as reliable as the annotated ORFs, validated by many different lines of evidence.

The overall support for ORFs derived from MS data appears to be variable across these genomes; therefore, we normalized the MS support degree of novel ORFs by calculating the ratio of MS support for novel ORFs to the support for annotated ORFs (MS support of novel ORF/MS support of annotated ORF) to enable intraspecies comparison. We found a higher normalized MS support for the novel ORFs identified from the yeast and *Arabidopsis* SNPs (Figure 6A and FigureS4A, see Supplementary Data available online at http://bib.oxfordjournals.org/). We compared the MS support to the ORFs predicted from SNPs and RPFs (Figure 6A and FigureS4A,see Supplementary Data available online at http://bib.oxfordjournals.org/). For the ORFs predicted from RPFs, the MS support for the novel ORFs was weaker than that for the annotated ORFs (Figure 6A and FigureS4A, see Supplementary Data available online at http://bib.oxfordjournals.org/). This suggests the novel ORFs predicted from SNPs are more reliable than those from RPFs, given that the former was supported to a degree nearly equivalent to that of annotated ORFs (Figure 6A). The normalized MS support also allowed a comparison of MS support between different categories of novel ORFs. Although substantial variation was observed across ORFs in different categories, the support for both uORFs and dORFs was generally comparable, both of which being somewhat higher than those for the annotated ORFs (Figure 6B and C and FigureS4B and C, see Supplementary Data available online at http://bib.oxfordjournals.org/).

We also performed this analysis to verify the ORFs identified from the SNPs of cotton (4 X) and wheat (6 X). Our data indicated that the novel ORFs identified from both the cotton and wheat SNPs were supported by MS data to a degree comparable to that of the annotated ORFs in the genome (Figure 6A). We could not compare the reliability of wheat novel ORFs identified from SNPs and RPFs because the latter is not yet available for wheat and cotton due to the short sequence lengths. However, our data show that the normalized MS support for cotton and wheat novel ORFs is stronger than that of yeast, *Arabidopsis* and rice novel ORFs identified from RPFs (Figure 6A and FigureS4A, see Supplementary Data available online at http://bib.oxfordjournals.org/).

Taken together, our data suggest that the novel ORFs identified from SNPs are as reliable as the annotated ORFs in either the simple or the complex genomes. Additionally, the reliability of novel ORFs identified from SNPs is higher than those identified from RPFs.

### ORF prediction independent of population size

**Figure 7:**
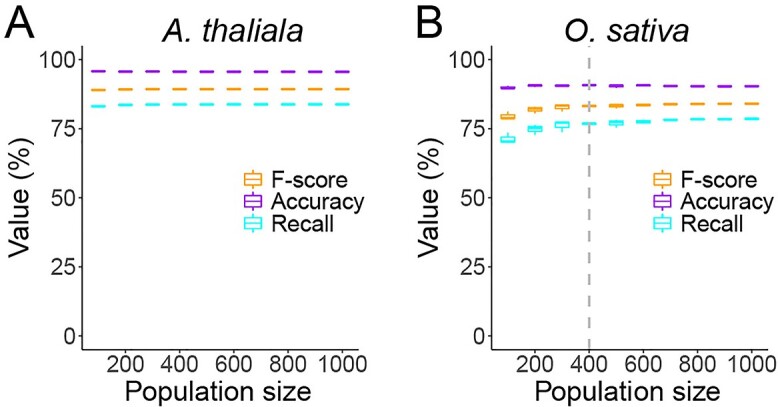
ORF predictions from SNPs are independent to the population size. Accessions were randomly sampled from the total SNP datasets of (**A**) *Arabidopsis* or (**B**) rice to generate subsets of SNPs with a population size ranging from 100 to 1000. The sampling and ORF predictions were repeated five times.

Given that most studied populations [48–52] are smaller than the ones tested in this work, we further explored whether SNP datasets from small populations can also be used to predict ORFs with acceptable accuracy. The datasets of *Arabidopsis* (1135 accessions) and rice (3024 accessions) were used in this test, from which 100 to 1000 accessions were randomly sampled and used for ORF prediction. Our results suggest that the performance (measured by F-score) of OrfPP was robust even when a small subset of *Arabidopsis* datasets was used (Figure 7A). The number of predicted ORFs generally increased as a function of population size but became saturated when the population size reached ~400 (Figure 7B) for rice. Although the recall rate was somewhat affected in small rice populations, the accuracy was independent of population size. Generally, the recall rates and accuracy of the ORFs predicted from SNPs are acceptable even for the smallest tested population (100 accessions). These results suggest that although the performance of OrfPP is somewhat compromised in small populations, it can be applied in most studied populations.

## Discussion

Synonymous codon mutations do not change the amino acid and are therefore subject to a more relaxed purification selection [53]. The third nucleotides in codons are wobble nucleotides that can change to other synonymous counterparts without changing the protein sequence, structure and function. Therefore, the third position of codons should have a higher diversity in the population, which would lead to a periodic increase in the nucleotide diversity every 3 nt along the CDSs. This characteristic formed the basis for identifying ORFs in the genome using SNPs.

### Application of OrfPP

In this work, we tested OrfPP in a total of 5 species representing fungi and plants, from haploid (*S. pombe*) to hexaploid (*T. aestivum*) species, with genome sizes ranging from ~12 Mb (*S. pombe*) to ~16000 Mb (*T. aestivum*). OrfPP successfully recovered most (~83%) of the known ORFs annotated in the reference genomes with considerable accuracy in all these tests, suggesting that the information recorded in SNP datasets can be a powerful ORF predictor. However, the predicted sORFs from SNPs appeared different from RPFs (Figure 4). In particular, dORFs were rarely predicted from RPFs, but many were predicted from SNPs, and only rare overlaps were found between the two methods. This difference could be explained by the fact that RPFs only capture the ORFs translated under the tested conditions, and the conflict between these two predictions implies potential biological roles of dORFs, although they are not usually translated. In fact, it is difficult to translate dORFs because ribosomes are usually stalled at stop codons and then released, so the translation of dORFs can probably only be initiated when stop-codon read-through occurs under some special conditions or by the new recruitment of ribosomes [54, 55]. Despite the divergence between the novel ORFs identified from SNPs and RPFs, evidence from MS data suggests comparable reliability for the annotated ORFs and the novel ORFs identified from either the RPFs or the SNPs (Figure 6).

Given the important biological roles of sORFs, we applied OrfPP to identify the sORFs in the polyploid plants, cotton and wheat, for which the RPF approach is not workable thus far due to the short lengths of RPFs. As a result, ~80% of the annotated ORFs in these genomes were successfully recovered and a total of 4704 and 5025 novel ORFs, including uORF and dORFs, were identified from cotton and wheat SNPs (Tables S3 and S4), respectively. As assessed by support from MS data, these novel ORFs are as reliable as the annotated ORFs in the genomes (Figure 6). Since identifying sORFs from RPFs is not yet feasible for polyploidy genomes, our work provides a good example showing that sORFs can be identified from SNPs. Additionally, the cotton and wheat sORFs provided here (Tables S3 and S4, see Supplementary Data available online at http://bib.oxfordjournals.org/) can be used in future works interested in the roles of sORFs in these crops.

### Comparison of RPF- and SNP-based approaches

Although RPFs have been proven powerful in predicting ORFs, several drawbacks have limited their application to a broader range of species. For example, RPFs can only be used to predict the translating ORFs, thus resulting in incomplete identification of ORFs. In addition, RPFs are too short to be correctly mapped to the loci where they originate, particularly in polyploid genomes. Lastly, the preparation of high-quality RPFs can be difficult, particularly in many non-model organisms. Although some quality-insensitive predictors have been developed to use low-quality RPFs [3, 23], RPFs with poor or no periodicity introduce unpredictable errors into the results and even lead to ORF prediction failure. Indeed, when we attempted to test OrfPP in many other species, such as *Solanum lycopersicum*, *S. pennelli* and *Medicago truncatula* [56], we found that the RPFs published in this study were not periodic and thus could not be utilized to predict ORFs. In contrast, nucleotide diversity accumulates during long-term genome evolution since the origin of this species so that this information can be used for genome-wide prediction. Compared to the problems of RPFs caused by their short size, populational SNPs are usually called by using 100 or 150 paired-ended reads, which are much longer than RPFs. Even for reads longer than 100 bp, incorrect or multiple mapping is also an inevitable problem in polyploidy or repetitive genomes, so special attention should be given to the interpretation of the results [47, 57]. It can be imagined that mapping RPFs with a size of 28 nt in these genomes would certainly be problematic. Furthermore, future application of long-read techniques in population studies would be helpful in finally solving the problems in SNP calling. Therefore, we propose that predicting ORFs from SNPs can be an alternative or supplementary approach to the existing methods and an efficient approach that can extend the study of sORFs to complex genomes, which cannot be currently achieved.

### Caution for the use of OrfPP

As OrfPP directly utilizes the periodicity of nucleotide diversities calculated from SNP datasets, any factors that can potentially affect the accuracy of SNPs would introduce mistakes into the final outputs.

(1) Low-quality reference genomeMore than 700 plant genomes have been released in recent decades, but many are of poor quality [19, 20]. The SNPs identified based on such low-quality reference genomes could contain many unpredictable mistakes.
(2) Mixture of SNPs from different speciesIn many studies, accessions from several, instead of only one, close species were included, but the SNPs were called based on only one reference [51, 58]. Mistakes could have been introduced when SNPs were called from the non-reference species.
(3) Autoploidy and repetitive genomesStudies of polyploid genomes are usually challenged by incorrect and multiple mapping problems caused by the short length of whole-genome sequencing reads. To address this problem, in some works, the authors remove the reads with multiple hits and use only the reads mapping to gene regions for further analyses [57]. However, polyploidy, particularly autoploid genomes, also has multiple and incorrect mapping problems in gene regions, affecting the predictions. With the decreasing cost of long reads, we believe these problems can eventually be solved when long reads are widely applied in population studies of these complex genomes.
(4) Young genomesORF prediction from SNPs relies heavily on nucleotide diversity in the population. Although our tests show considerable independence from population size, the prediction could be incomplete for young genomes because only a few nucleotide substitutions have accumulated since the divergence of these genomes.

## Usage OF OrfPP

OrfPP has been distributed to the Python Package Index (https://pypi.org/project/OrfPP/1.0/) and can be easily installed using pip tools. Three compulsory inputs are needed: genome sequence (—genome), genome annotation (—gtf) and populational nucleotide diversity (—pi). The diversity of nucleotides at each position can be calculated using vcftools [58] with the command ‘vcftools --gzvcf SNPs.vcf.gz --site-pi --out output.pi’. The other five options can be customized. For example, OrfPP allows ORFs starting with noncanonical initiation codons to facilitate the prediction of ORFs initiated by alternative start codons. An option of ‘—nCores’ is implemented to use multiple processors to speed up the processing of data, which might be required to deal with genomes of enormous size.

**Key Points**

- Population nucleotide diversity shows that a 3-nt periodicity can be used to predict open reading frames (ORFs).
- A python package ‘OrfPP’ is developed to predict ORFs from single-nucleotide polymorphisms datasets.
- Application ‘OrfPP’ in polyploidy genomes recovered ~83% of the annotated ORFs.
- OrfPP could help extend the studies of small ORFs to organisms with complex genomes.

## Supplementary Material

Figure_S1_bbac210

Figure_S3_bbac210

Figure_S4_bbac210

Table_S3_bbac210

Table_S4_bbac210

## Notes

**Mengyun Jiang** is a graduate student at the Chinese Academy of Agricultural Sciences and Henan University, China. She is interested in the development of bioinformatics pipelines.

**Weidong Ning** is a graduate student at the Chinese Academy of Agricultural Sciences and Huazhong Agricultural University, China. He is interested in the analysis of population genomic datasets.

**Shishi Wu** is a graduate student at the Chinese Academy of Agricultural Sciences and Henan University, China. She is interested in the secondary analysis of public datasets.

**Xingwei Wang** is a graduate student at the Chinese Academy of Agricultural Sciences and Henan University, China. His research interest is visualization of omics datasets.

**Kun Zhu** is a graduate student at the Chinese Academy of Agricultural Sciences and Henan University, China. His research interest is machine learning.

**Aomei Li** is a visitor to the Chinese Academy of Agricultural Sciences. She is interested in a genomic comparison between complex genomes.

**Yongyao Li** is a technician at the Chinese Academy of Agricultural Sciences. He is interested in data analysis and visualization.

**Shifeng Cheng** is a professor at the Chinese Academy of Agricultural Sciences. He is interested in comparative genomic studies and plant genome evolution.

**Bo Song** is an associate professor at the Chinese Academy of Agricultural Sciences. His research interests include the development of bioinformatics pipelines, comparison and analysis of omics datasets and plant genome evolution.

## References

[1] Calviello L, Mukherjee N, Wyler E, et al. Detecting actively translated open reading frames in ribosome profiling data. Nat Methods 2016;13:165–70. 26657557 10.1038/nmeth.3688

[2] Calviello L, Ohler U. Beyond read-counts: ribo-seq data analysis to understand the functions of the transcriptome. Trends Genet 2017;33:728–44. 28887026 10.1016/j.tig.2017.08.003

[3] Song B, Jiang M, Gao L. RiboNT: a noise-tolerant predictor of open reading frames from ribosome-protected footprints. Life (Basel) 2021;11:701. 34357073 10.3390/life11070701 PMC8307163

[4] Spealman P, Naik AW, May GE, et al. Conserved non-AUG uORFs revealed by a novel regression analysis of ribosome profiling data. Genome Res 2018;28:214–22. 29254944 10.1101/gr.221507.117 PMC5793785

[5] Xiao Z, Huang R, Xing X, et al. De novo annotation and characterization of the translatome with ribosome profiling data. Nucleic Acids Res 2018;46:e61. 29538776 10.1093/nar/gky179 PMC6007384

[6] Bazin J, Baerenfaller K, Gosai SJ, et al. Global analysis of ribosome-associated noncoding RNAs unveils new modes of translational regulation. Proc Natl Acad Sci U S A 2017;114:E10018–27. 29087317 10.1073/pnas.1708433114 PMC5699049

[7] Merino-Valverde I, Greco E, Abad M. The microproteome of cancer: From invisibility to relevance. Exp Cell Res 2020;392:111997. 32302626 10.1016/j.yexcr.2020.111997

[8] Jayaram DR, Frost S, Argov C, et al. Unraveling the hidden role of a uORF-encoded peptide as a kinase inhibitor of PKCs. Proc Natl Acad Sci U S A 2021;118:e2018899118. 34593629 10.1073/pnas.2018899118 PMC8501901

[9] Gao X, Wan J, Liu B, et al. Quantitative profiling of initiating ribosomes in vivo. Nat Methods 2015;12:147–53. 25486063 10.1038/nmeth.3208 PMC4344187

[10] Hayden CA, Jorgensen RA. Identification of novel conserved peptide uORF homology groups in Arabidopsis and rice reveals ancient eukaryotic origin of select groups and preferential association with transcription factor-encoding genes. BMC Biol 2007;5:32. 17663791 10.1186/1741-7007-5-32 PMC2075485

[11] Hsu PY, Calviello L, Wu HL, et al. Super-resolution ribosome profiling reveals unannotated translation events in Arabidopsis. Proc Natl Acad Sci U S A 2016;113:E7126–35. 27791167 10.1073/pnas.1614788113 PMC5111709

[12] Ingolia NT, Brar GA, Rouskin S, et al. The ribosome profiling strategy for monitoring translation in vivo by deep sequencing of ribosome-protected mRNA fragments. Nat Protoc 2012;7:1534–50. 22836135 10.1038/nprot.2012.086 PMC3535016

[13] Ingolia NT, Lareau LF, Weissman JS. Ribosome profiling of mouse embryonic stem cells reveals the complexity and dynamics of mammalian proteomes. Cell 2011;147:789–802. 22056041 10.1016/j.cell.2011.10.002 PMC3225288

[14] Juntawong P, Girke T, Bazin J, et al. Translational dynamics revealed by genome-wide profiling of ribosome footprints in Arabidopsis. Proc Natl Acad Sci U S A 2014;111:E203–12. 24367078 10.1073/pnas.1317811111 PMC3890782

[15] Lee S, Liu B, Lee S, et al. Global mapping of translation initiation sites in mammalian cells at single-nucleotide resolution. Proc Natl Acad Sci U S A 2012;109:E2424–32. 22927429 10.1073/pnas.1207846109 PMC3443142

[16] Wang H, Wang Y, Xie Z. Computational resources for ribosome profiling: from database to Web server and software. Brief Bioinform 2019;20:144–55. 28968766 10.1093/bib/bbx093

[17] Andreev DE, O'Connor PB, Loughran G, et al. Insights into the mechanisms of eukaryotic translation gained with ribosome profiling. Nucleic Acids Res 2017;45:513–26. 27923997 10.1093/nar/gkw1190 PMC5314775

[18] Heyer EE, Moore MJ. Redefining the translational status of 80S monosomes. Cell 2016;164:757–69. 26871635 10.1016/j.cell.2016.01.003

[19] Marks RA, Hotaling S, Frandsen PB, et al. Representation and participation across 20 years of plant genome sequencing. Nat Plants 2021;7:1571–8. 34845350 10.1038/s41477-021-01031-8 PMC8677620

[20] Sun Y, Shang L, Zhu QH, et al. Twenty years of plant genome sequencing: achievements and challenges. Trends Plant Sci 2021;27:391–401. 34782248 10.1016/j.tplants.2021.10.006

[21] Li S, Le B, Ma X, et al. Biogenesis of phased siRNAs on membrane-bound polysomes in Arabidopsis. Elife 2016;5:e22750. 27938667 10.7554/eLife.22750 PMC5207768

[22] Reid DW, Shenolikar S, Nicchitta CV. Simple and inexpensive ribosome profiling analysis of mRNA translation. Methods 2015;91:69–74. 26164698 10.1016/j.ymeth.2015.07.003 PMC4684803

[23] Xu Z, Hu L, Shi B, et al. Ribosome elongating footprints denoised by wavelet transform comprehensively characterize dynamic cellular translation events. Nucleic Acids Res 2018;46:e109. 29945224 10.1093/nar/gky533 PMC6182183

[24] Calviello L, Hirsekorn A, Ohler U. Quantification of translation uncovers the functions of the alternative transcriptome. Nat Struct Mol Biol 2020;27:717–25. 32601440 10.1038/s41594-020-0450-4

[25] Choudhary S, Li W, A DS. Accurate detection of short and long active ORFs using Ribo-seq data. Bioinformatics 2020;36:2053–9. 31750902 10.1093/bioinformatics/btz878 PMC7141849

[26] Yang XY, Song B, Cui J, et al. Comparative ribosome profiling reveals distinct translational landscapes of salt-sensitive and -tolerant rice. BMC Genomics 2021;22:612. 34384368 10.1186/s12864-021-07922-6 PMC8359061

[27] Rahim KJ, Burr WS, Thomson DJ. Appendix A: multitaper R package in applications of multitaper spectral analysis to nonstationary data. Queen's University, Kingston, ON, Canada, 2014;149–83.

[28] Kim D, Paggi JM, Park C, et al. Graph-based genome alignment and genotyping with HISAT2 and HISAT-genotype. Nat Biotechnol 2019;37:907–15. 31375807 10.1038/s41587-019-0201-4 PMC7605509

[29] Taglini F, Chapman E, van Nues R, et al. Mkt1 is required for RNAi-mediated silencing and establishment of heterochromatin in fission yeast. Nucleic Acids Res 2020;48:1239–53. 31822915 10.1093/nar/gkz1157 PMC7026591

[30] Kretzschmar FK, Mengel LA, Muller AO, et al. PUX10 Is a lipid droplet-localized scaffold protein that interacts with CELL DIVISION CYCLE48 and is involved in the degradation of lipid droplet proteins. Plant Cell 2018;30:2137–60. 30087207 10.1105/tpc.18.00276 PMC6181012

[31] Hamzelou S, Kamath KS, Masoomi-Aladizgeh F, et al. Wild and cultivated species of rice have distinctive proteomic responses to drought. Int J Mol Sci 2020;21:5980. 10.3390/ijms21175980 PMC7504292 32825202

[32] Xiao S, Liu L, Zhang Y, et al. Tandem mass tag-based (TMT) quantitative proteomics analysis reveals the response of fine roots to drought stress in cotton (*Gossypium hirsutum L.*). BMC Plant Biol 2020;20:328. 10.1186/s12870-020-02531-z PMC7353779 32652934

[33] Ghatak A, Chaturvedi P, Bachmann G, et al. Physiological and proteomic signatures reveal mechanisms of superior drought resilience in pearl millet compared to wheat. Front Plant Sci 2020;11:600278. 33519854 10.3389/fpls.2020.600278 PMC7838129

[34] Cox J, Mann M. MaxQuant enables high peptide identification rates, individualized p.p.b.-range mass accuracies and proteome-wide protein quantification. Nat Biotechnol 2008;26:1367–72. 19029910 10.1038/nbt.1511

[35] Altshuler D, Durbin RM, Abecasis GR, et al. A map of human genome variation from population-scale sequencing. Nature 2010;467:1061–73. 20981092 10.1038/nature09534 PMC3042601

[36] Altshuler DM, Durbin RM, Abecasis GR, et al. An integrated map of genetic variation from 1,092 human genomes. Nature 2012;491:56–65. 23128226 10.1038/nature11632 PMC3498066

[37] Genomes Consortium . Electronic address mngoaa, Genomes C. 1,135 Genomes reveal the global pattern of polymorphism in *Arabidopsis thaliana*. Cell 2016;166:481–91. 27293186 10.1016/j.cell.2016.05.063 PMC4949382

[38] Jeffares DC, Jolly C, Hoti M, et al. Transient structural variations have strong effects on quantitative traits and reproductive isolation in fission yeast. Nat Commun 2017;8:14061. 28117401 10.1038/ncomms14061 PMC5286201

[39] Wang W, Mauleon R, Hu Z, et al. Genomic variation in 3,010 diverse accessions of Asian cultivated rice. Nature 2018;557:43–9. 29695866 10.1038/s41586-018-0063-9 PMC6784863

[40] Ikemura T . Codon usage and tRNA content in unicellular and multicellular organisms. Mol Biol Evol 1985;2:13–34. 3916708 10.1093/oxfordjournals.molbev.a040335

[41] Kanaya S, Yamada Y, Kinouchi M, et al. Codon usage and tRNA genes in eukaryotes: correlation of codon usage diversity with translation efficiency and with CG-dinucleotide usage as assessed by multivariate analysis. J Mol Evol 2001;53:290–8. 11675589 10.1007/s002390010219

[42] Liu CZ, Yuan JB, Zhang XJ, et al. tRNA copy number and codon usage in the sea cucumber genome provide insights into adaptive translation for saponin biosynthesis. Open Biol 2021;11:210190. 34753322 10.1098/rsob.210190 PMC8580430

[43] Aspden JL, Eyre-Walker YC, Phillips RJ, et al. Extensive translation of small Open Reading Frames revealed by Poly-Ribo-Seq. Elife 2014;3:e03528. 25144939 10.7554/eLife.03528 PMC4359375

[44] Erhard F, Halenius A, Zimmermann C, et al. Improved Ribo-seq enables identification of cryptic translation events. Nat Methods 2018;15:363–6. 29529017 10.1038/nmeth.4631 PMC6152898

[45] Li J, Yuan D, Wang P, et al. Cotton pan-genome retrieves the lost sequences and genes during domestication and selection. Genome Biol 2021;22:119. 33892774 10.1186/s13059-021-02351-w PMC8063427

[46] Cheng H, Liu J, Wen J, et al. Frequent intra- and inter-species introgression shapes the landscape of genetic variation in bread wheat. Genome Biol 2019;20:136. 31300020 10.1186/s13059-019-1744-x PMC6624984

[47] Zhou Y, Zhao XB, Li YW, et al. Triticum population sequencing provides insights into wheat adaptation. Nat Genet 2020;52:1412–22. 33106631 10.1038/s41588-020-00722-w

[48] Cui J, Yang Y, Luo S, et al. Whole-genome sequencing provides insights into the genetic diversity and domestication of bitter gourd (Momordica spp.). Hortic Res 2020;7:85. 32528697 10.1038/s41438-020-0305-5 PMC7261802

[49] Duan N, Bai Y, Sun H, et al. Genome re-sequencing reveals the history of apple and supports a two-stage model for fruit enlargement. Nat Commun 2017;8:249. 28811498 10.1038/s41467-017-00336-7 PMC5557836

[50] Li X, Yang J, Shen M, et al. Whole-genome resequencing of wild and domestic sheep identifies genes associated with morphological and agronomic traits. Nat Commun 2020;11:2815. 32499537 10.1038/s41467-020-16485-1 PMC7272655

[51] Song B, Song Y, Fu Y, et al. Draft genome sequence of Solanum aethiopicum provides insights into disease resistance, drought tolerance and the evolution of the genome. Gigascience 2019;8:giz115. 31574156 10.1093/gigascience/giz115 PMC6771550

[52] Zhang Z, Jia Y, Almeida P, et al. Whole-genome resequencing reveals signatures of selection and timing of duck domestication. Gigascience 2018;7:giy027. 10.1093/gigascience/giy027 PMC6007426 29635409

[53] Hurst LD . The Ka/Ks ratio: diagnosing the form of sequence evolution. Trends Genet 2002;18:486. 12175810 10.1016/s0168-9525(02)02722-1

[54] Nobuta R, Machida K, Sato M, et al. eIF4G-driven translation initiation of downstream ORFs in mammalian cells. Nucleic Acids Res 2020;48:10441–55. 32941651 10.1093/nar/gkaa728 PMC7544200

[55] Wu Q, Wright M, Gogol MM, et al. Translation of small downstream ORFs enhances translation of canonical main open reading frames. EMBO J 2020;39:e104763. 32744758 10.15252/embj.2020104763 PMC7459409

[56] Reynoso MA, Kajala K, Bajic M, et al. Evolutionary flexibility in flooding response circuitry in angiosperms. Science 2019;365:1291–5. 31604238 10.1126/science.aax8862 PMC7710369

[57] Todesco M, Owens GL, Bercovich N, et al. Massive haplotypes underlie ecotypic differentiation in sunflowers. Nature 2020;584:602–7. 32641831 10.1038/s41586-020-2467-6

[58] Cai X, Sun X, Xu C, et al. Genomic analyses provide insights into spinach domestication and the genetic basis of agronomic traits. Nat Commun 2021;12:7246. 34903739 10.1038/s41467-021-27432-z PMC8668906

[59] Danecek P, Auton A, Abecasis G, et al. The variant call format and VCFtools. Bioinformatics 2011;27:2156–8. 21653522 10.1093/bioinformatics/btr330 PMC3137218

