# Supplementary figures and images for: Three-nucleotide periodicity of nucleotide diversity in a population enables the identification of open reading frames
Source: Brief Bioinform. 2022 Jun 13;23(4):bbac210. doi: 10.1093/bib/bbac210 (PMC9294425; doi:10.1093/bib/bbac210)

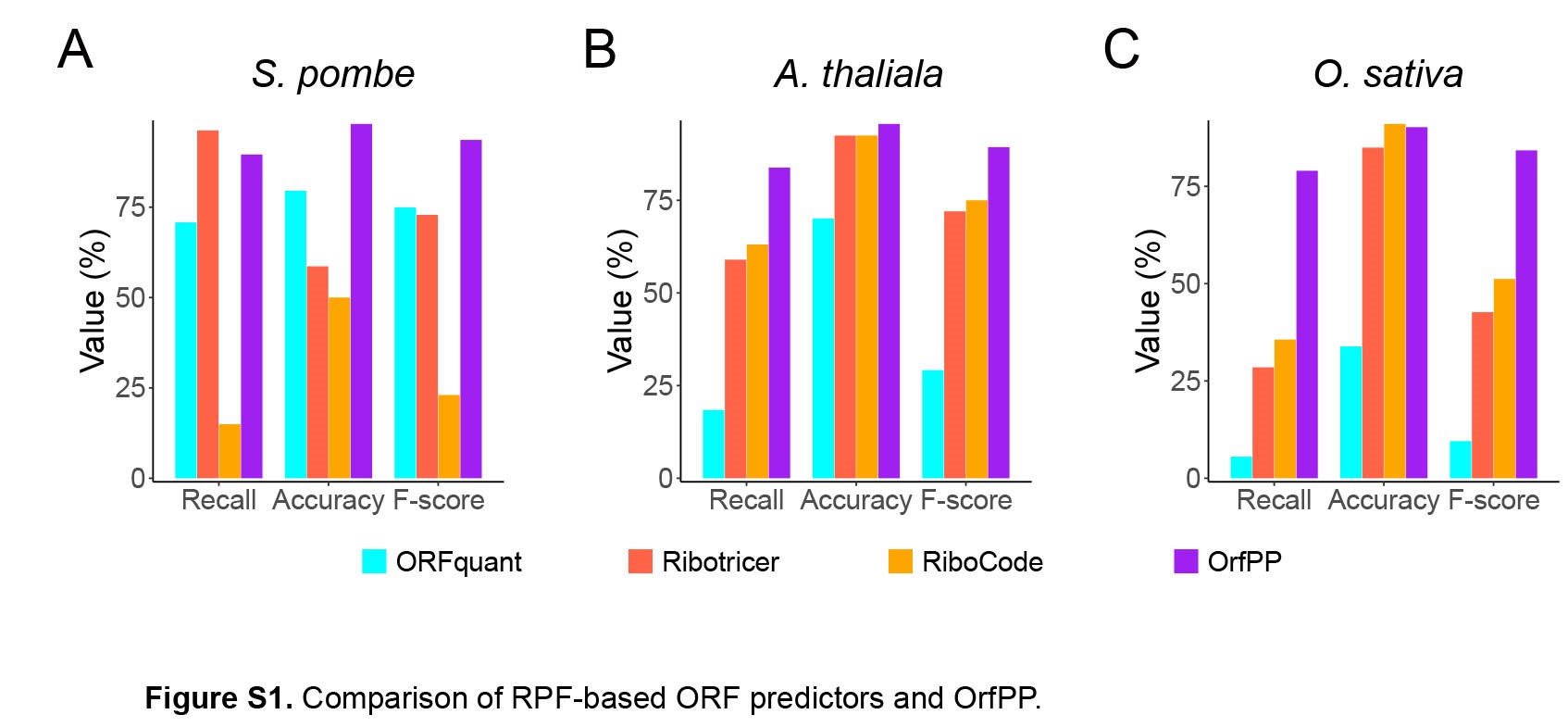

Supplement: Figure_S1_bbac210 [file figure_s1_bbac210.jpeg]

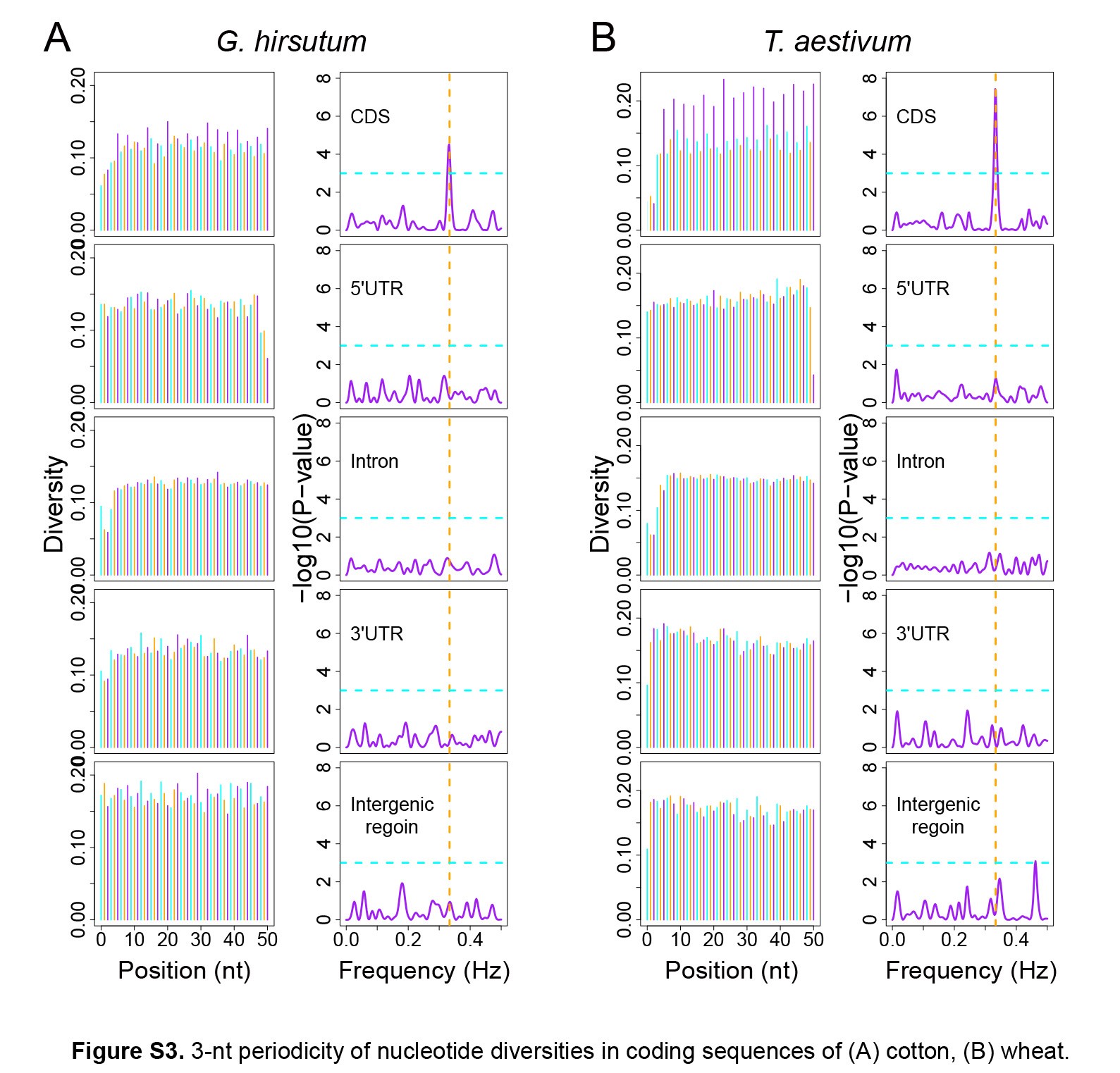

Supplement: Figure_S3_bbac210 [file figure_s3_bbac210.jpeg]

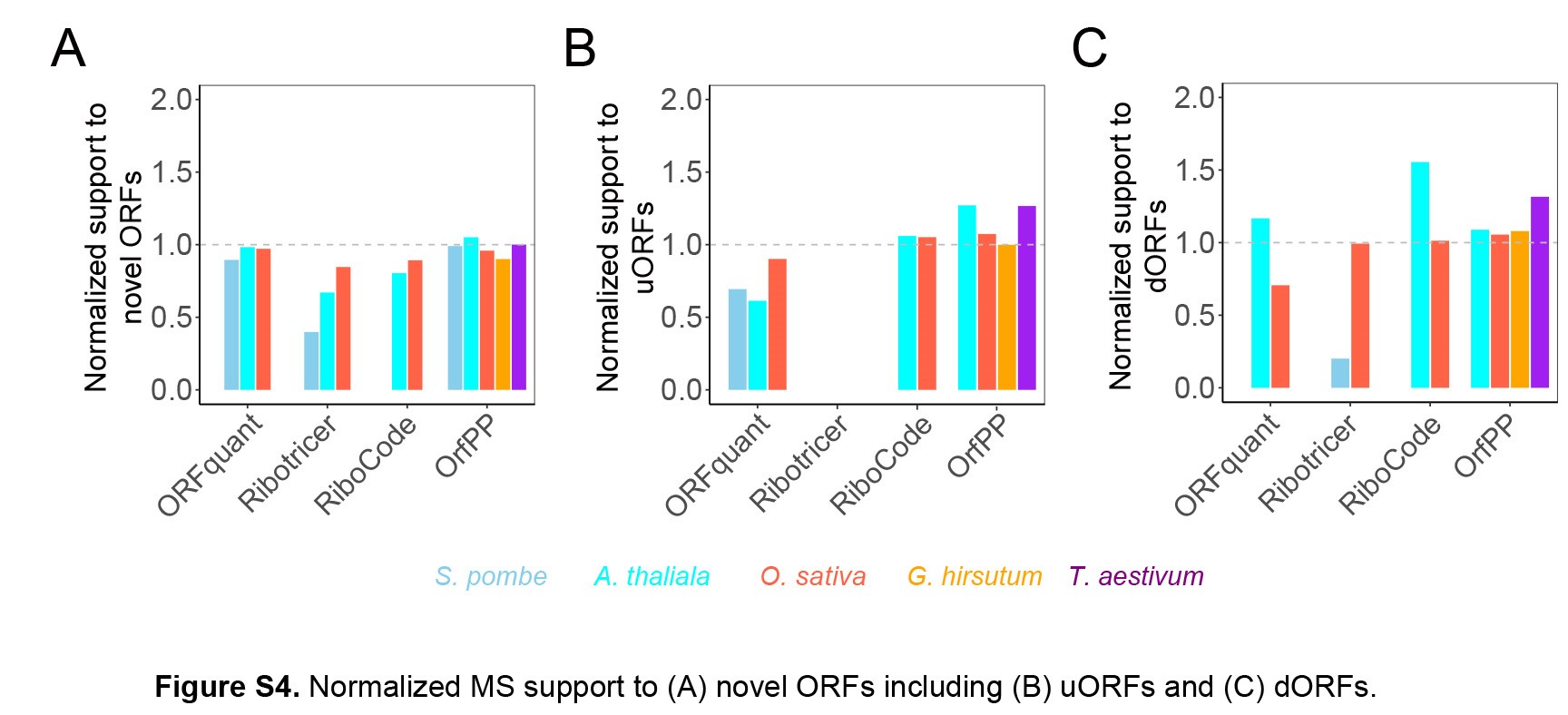

Supplement: Figure_S4_bbac210 [file figure_s4_bbac210.jpeg]
